# SkewIT: The Skew Index Test for large-scale GC Skew analysis of bacterial genomes

**DOI:** 10.1371/journal.pcbi.1008439

**Published:** 2020-12-04

**Authors:** Jennifer Lu, Steven L. Salzberg

**Affiliations:** 1 Department of Biomedical Engineering, Johns Hopkins University, Baltimore, Maryland, United States; 2 Center for Computational Biology, Whiting School of Engineering, Johns Hopkins University, Baltimore, Maryland, United States; 3 Departments of Computer Science and Biostatistics, Johns Hopkins University, Baltimore, Maryland, United States; University of Chicago, UNITED STATES

## Abstract

GC skew is a phenomenon observed in many bacterial genomes, wherein the two replication strands of the same chromosome contain different proportions of guanine and cytosine nucleotides. Here we demonstrate that this phenomenon, which was first discovered in the mid-1990s, can be used today as an analysis tool for the 15,000+ complete bacterial genomes in NCBI’s Refseq library. In order to analyze all 15,000+ genomes, we introduce a new method, SkewIT (Skew Index Test), that calculates a single metric representing the degree of GC skew for a genome. Using this metric, we demonstrate how GC skew patterns are conserved within certain bacterial phyla, e.g. Firmicutes, but show different patterns in other phylogenetic groups such as Actinobacteria. We also discovered that outlier values of SkewIT highlight potential bacterial mis-assemblies. Using our newly defined metric, we identify multiple mis-assembled chromosomal sequences in previously published complete bacterial genomes. We provide a SkewIT web app https://jenniferlu717.shinyapps.io/SkewIT/ that calculates SkewI for any user-provided bacterial sequence. The web app also provides an interactive interface for the data generated in this paper, allowing users to further investigate the SkewI values and thresholds of the Refseq-97 complete bacterial genomes. Individual scripts for analysis of bacterial genomes are provided in the following repository: https://github.com/jenniferlu717/SkewIT.

## Author summary

Even though every guanine (G) is paired with a cytosine (C) in double-stranded DNA molecules, bacterial genomes have more G’s than C’s when we focus only on a single strand in the direction of replication, called the leading strand. This phenomenon, called GC skew, is so ubiquitous that it has been used reliably to identify the replication origin (the location from which DNA begins the process of replicating itself) in thousands of bacteria. Here we describe a new method that automatically captures the “skewness” of a genome by finding the origin and terminus of replication, and then reporting skewness as a single number. We calculated this value for over 15,000 genomes, and found that most phylogenetic groups have a characteristic amount of skewness. We also observed that an unusually low value for skewness sometimes indicated that the genome was incorrectly assembled. To assist others in this type of analysis, we developed a graphical tool to compute and display GC-skew for any genome of interest.

This is a *PLOS Computational Biology* Methods paper.

## Introduction

Two of the largest and most widely-used nucleotide databases are GenBank [1], which has been a shared repository for more than 25 years (and which is mirrored by the EMBL and DDBJ databases [1, 2]), and RefSeq, a curated subset of GenBank [2]. For sequences to be entered into RefSeq, curators at NCBI perform both automated and manual checks to ensure minimal contamination and high sequence quality. Despite these efforts, multiple studies have identified contamination in RefSeq and other publicly available genome databases [3–7]. NCBI requires Refseq assemblies to have an appropriate genome length as compared to existing genomes from the same species, and it labels assemblies as “complete” if the genome exists in one contiguous sequence per chromosome, with no unplaced scaffolds and with all chromosomes present. However, NCBI does not perform additional checks, most of which would be computationally expensive, to ensure that a genome sequence was assembled correctly. In this study, we propose a new method, SkewIT (Skew Index Test), for validating bacterial genome assemblies based on the phenomenon of GC-skew. We applied this method to 15,067 complete bacterial genomes in RefSeq, identifying many potential misassemblies as well as trends in GC-skew that are characteristic of some bacterial clades.

### Bacterial GC skew

GC skew is a non-homogeneous distribution of nucleotides in bacterial DNA strands first discovered in the mid-1990s [8, 9]. Although double-stranded DNA must contain precisely equal numbers of cytosine (C) and guanine (G) bases, the distribution of these nucleotides along a single strand in bacterial chromosomes may be asymmetric. Analysis of many bacterial chromosomes has revealed two distinct compartments, one that is more G-rich and the other that is more C-rich.

Most bacterial genomes are organized into single, circular chromosomes. Replication of the circular chromosomes begins at a single point known as the origin of replication (*ori*) and proceeds bidirectionally until reaching the replication terminus (*ter*). Because the replication process only adds DNA nucleotides to the 3’ end of a DNA strand, it must use two slightly different DNA synthesis methods to allow bidirectional replication of the circular chromosome. The leading strand is synthesized continuously from the 5’ to 3’ end. The lagging strand, in contrast, is synthesized by first creating small Okazaki DNA fragments [10] that are then added to the growing strand in the 3’ to 5’ direction.

These two slightly different replication processes lead to different mutational biases. Notably, the DNA polymerase replicating the leading strand has a higher instance of hydrolytic deamination of the cytosine, resulting in C→ T (thymine) mutations [11]. However, the replication mechanisms for the lagging strand have a higher instance of repair of the same C→ T mutation [12]. These differences between the leading and lagging strands result in GC-skew, where the leading strand contains more Gs than Cs, while the lagging strand has more Cs than Gs.

Linear bacterial genomes also exhibit GC skew despite the difference in genome organization. For example, DNA replication of *Borrelia burgdorferi* begins at the center of the linear chromosome and proceeds bidirectionally until reaching the chromosome ends [13, 14]. This bidirectional replication shows the same GC-skew pattern seen on circular chromosomes.

### Quantitative measurements of GC skew

Since the 1990s, GC skew has been used as a quantitative measure of the guanine and cytosine distribution along a genome sequence, where GC skew is computed using the formula (G-C)/(G+C), where G is the number of guanines and C is the number of cytosines in a fixed-size window [9]. GC skew plots are generated by calculating GC skew in adjacent or overlapping windows across the full length of a bacterial genome [8]. Analysis of these plots confirmed the separation of many bacterial genomes into a leading strand with largely positive GC skew and a lagging strand with negative GC skew. The GC skew effect is strong enough that it can be used to identify, within a few kilobases, the *ori/ter* locations.

GC skew plots then evolved into cumulative skew diagrams, which sum the GC skew value in adjacent windows along the bacterial genome [9]. These diagrams sometimes allow more precise identification of the *ori/ter* locations, where the origin is located at the global minimum and the terminus is at the global maximum.

### GC skew applications and analyses

Over the last two decades, researchers have employed both GC skew and cumulative GC skew (CGS) diagrams to analyze bacterial genomes. Initial studies confirmed that GC skew was a strong indicator of the direction of replication in the genomes of *Escherichia coli* [15], *Bacillus subtilis*, *Haemophilus influenzae*, and *Borrelia burgdorferi* [8]. In 1998, Mclean et. al. compared GC skew among 9 bacterial genomes and 3 archaeal genomes, revealing strong GC skew in all 9 bacteria but weak or no GC skew signals in the archaeal genomes [16]. In 2002, Rocha et. al. used CGS to predict ori/ter locations for 15 bacterial genomes [17] and in 2017, Zhang et. al. analyzed GC skew across more than 2000 bacterial genomes [18].

Although GC skew has been used as an indicator of the replication strand in thousands of bacterial genomes, it is rarely used as a means to validate genome assemblies. However, the association between GC skew and replication is strong enough that when a genome has a major mis-assembly such as a translocation or inversion, the GC skew plot is clearly disrupted [19]. While existing mis-assembly detection methods (e.g. QUAST [20], REAPR [21], misFinder [22]) require the reads used in genome assembly and/or a reference sequence, GC skew can indicate a potential mis-assembly from the genome sequence alone.

In this paper, we introduce SkewIT (Skew Index Test) as an efficient method to calculate the degree of GC skew in a genome. The SkewIT test allows us to quickly analyze all 15,000+ complete bacterial genomes in NCBI’s RefSeq library by assigning each genome a single SkewI (Skew Index) value representing the degree of GC skew. We then use the SkewI value to compare GC skew across bacterial clades without requiring GC skew or CGS diagrams. Below we demonstrate how the degree of GC skew tends to be conserved within certain bacterial taxa; e.g. *Klebsiella* species have high values of the SkewI, while *Bordetella* have much lower values. During this analysis, we discovered that bacterial genomes with outlier values of SkewIT are highly likely to contain mis-assemblies. Using our newly defined metric, we identify multiple potentially mis-assembled chromosomal sequences in the Refseq library of complete bacterial genomes.

## Materials and methods

**Fig 1:**
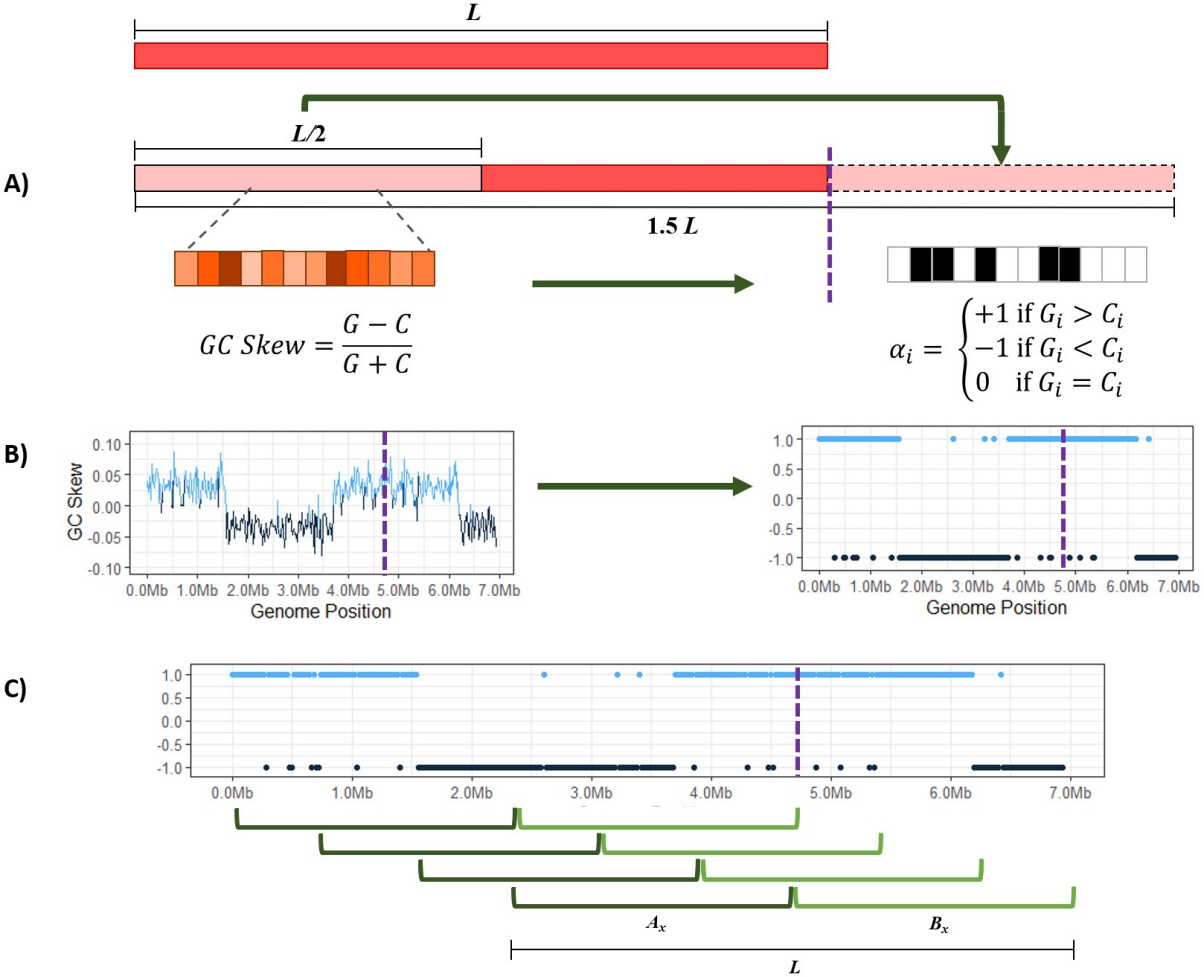
The SkewIT algorithm. A genome of length L is“circularized” by taking the first half of the sequence (L/2) and concatenating that sequence onto the end of the genome (A). The algorithm then splits the sequence into many shorter windows of length *w*. We assign each window an *α* value [1,-1,0] based on whether there are more Gs, Cs, or equal quantities of both. (B) The GC skew statistic is shown (left) plotted across the E. coli genome, with a purple dotted line showing where the original sequence ended, prior to concatenating 1/2 of the genome to the end. The plot on the right shows the *α* value plotted for the same genome. (C) SkewIT finds the location in the genome with the greatest difference in GC skew between the first half and the second half of the genome, by using a pair of sliding windows to find the greatest sum of differences between the *α* values for the two halves.

SkewIT quantifies GC skew patterns by assigning a single value between 0 and 1 to the complete chromosomal sequence of a bacterial genome, where higher values indicate greater GC skew, and lower values indicate that no GC skew pattern was detected. Fig 1 illustrates the overall method.

Although many published bacterial genome assemblies set the start of the published assembly (i.e., position 1) at the origin of replication, many other bacterial genomes set coordinate 1 arbitrarily. (Because the genomes are circular, there is no unambiguous choice for the beginning of the sequence. DNA databases only contain linear sequences, and therefore some coordinate must be chosen as position 1.) Therefore, we first “circularize” each bacterial genome of size *L* by appending the first *L*/2 bases of the genome to the end, resulting in a sequence length of 1.5*L* (Fig 1A). This ensures that the full genome starting from the origin of replication will be contained within one of the subsequences of length L between positions 0 and L/2.

Next, we select a GC skew window size *w* and split the genome into 1.5*L*/*w* adjacent windows; e.g., for a 1-megabase genome with a 10-Kb window length, we would create 150 windows. In each window *i* ∈ [1, 2, ⋯1.5*L*/*w*], we count the frequency of guanine (G) and cytosine (C) bases. Traditionally, GC skew was calculated for each window using Eq (1):

$$\begin{array}{c} \text{GC-Skew}=\frac{G-C}{G+C} \end{array}$$ (1)

Although the GC skew formula accounts for the relative quantities of G and C bases, our method only evaluates which base is more prominent in each window. Fig 1B demonstrates how we convert the GC skew formula into a simplified version that instead assigns each window a score *α*_*i*_ using Eq (2):

$$\begin{array}{c} \alpha_{i}=\{\begin{array}{cc} +1 & \text{if}\;G_{i}>C_{i}\\ -1 & \text{if}\;G_{i}<C_{i}\\ 0 & \text{if}\;G_{i}=C_{i} \end{array} \end{array}$$ (2)

We evaluate the “skewness” of the genome using a sliding window of size *L*, sliding over one window width at a time. Each window *x* ∈ [1, 2, ⋯0.5*L*/*w*] is first split into two equal partitions that each cover 50% of the original genome. We then calculate the sum the *α*_*i*_ values for each partition and determine the absolute difference in sum of GC Skew values between the partitions as shown in Eq (3) and Fig 1C:

$$\begin{array}{c} |A_{x}-B_{x}|=|\sum_{i=x}^{x+L/2w}\alpha_{i}-\sum_{i=x+L/2w}^{x+L/w}\alpha_{i}| \end{array}$$ (3)

*A*_*x*_ is the sum of the *α* values within the partition, and *B*_*x*_ is the sum of the *α* values for the second partition. For example, Eq (4) shows how we calculate |*A*_1_ − *B*_1_|, the skewness for the first sliding window from a genome.

$$\begin{array}{c} \left|A-B\right|=|\sum_{i=1}^{L/2w}\alpha_{i}-\sum_{i=L/2w}^{L/w}\alpha_{i}| \end{array}$$ (4)

Then, in order to allow for the leading and lagging strands to be slightly different in length, we move the transition point between the two partitions a small distance (4% of the genome length by default) to the left and right, allowing the leading strand to be anywhere between 46% and 54% of the genome length, and recalculating the difference in sums of *α* values. The transition point is chosen to maximize |*A*_*x*_ − *B*_*x*_| for this window.

Finally, we determine the maximum value of |*A*_*x*_ − *B*_*x*_|, which gives us the window where the greatest difference exists between the GC content of the two partitions of the genome. In order to be provide a consistent value between 0 and 1 despite genome length *L* or window size *w*, we define the skew index (*SkewI*) as the following normalized value:

$$\begin{array}{c} SkewI=\frac{w}{L}\text{max}\left|A_{x}-B_{x}\right| \end{array}$$ (5)

### Software availability

The SkewIT program is available at https://jenniferlu717.shinyapps.io/SkewIT/ as an interactive web app which calculates SkewI and plots GC Skew from a user-provided bacterial genome FASTA file. The app additionally provides users with an interactive interface to explore the data presented here across all bacterial genomes or for individual bacterial genera. Additional information about the SkewIT web application is provided in the S1 Text file and S6–S8 Figs. SkewIT is also available as individual executable scripts at https://github.com/jenniferlu717/SkewIT.

## Results and discussion

We applied the SkewIT method to the complete bacterial genomes from NCBI RefSeq Release 97 (released on November 4, 2019). We only evaluated bacterial chromosomes that were > 50,000bp in length and excluded plasmids from this analysis. In total, we tested 15,067 genomes representing 4,471 species and 1,148 genera.

First, we compared SkewI values using the various window sizes *w* of 10Kb, 15Kb, 20Kb, 25Kb, and 30Kb (S1 Fig). From our analysis, smaller window sizes (10Kb and 15Kb) caused the SkewI values across all bacterial genomes to be lower, as SkewI was more sensitive to local fluctuations in polarity. However, as window sizes become too large, we were no longer able to accurately calculate SkewI for smaller genomes. Therefore, we selected a window size of 20Kb across all genomes tested. S1 Table lists each genome with their SkewI values. The table also provides the main taxonomy assignments for each genome.

**Fig 2:**
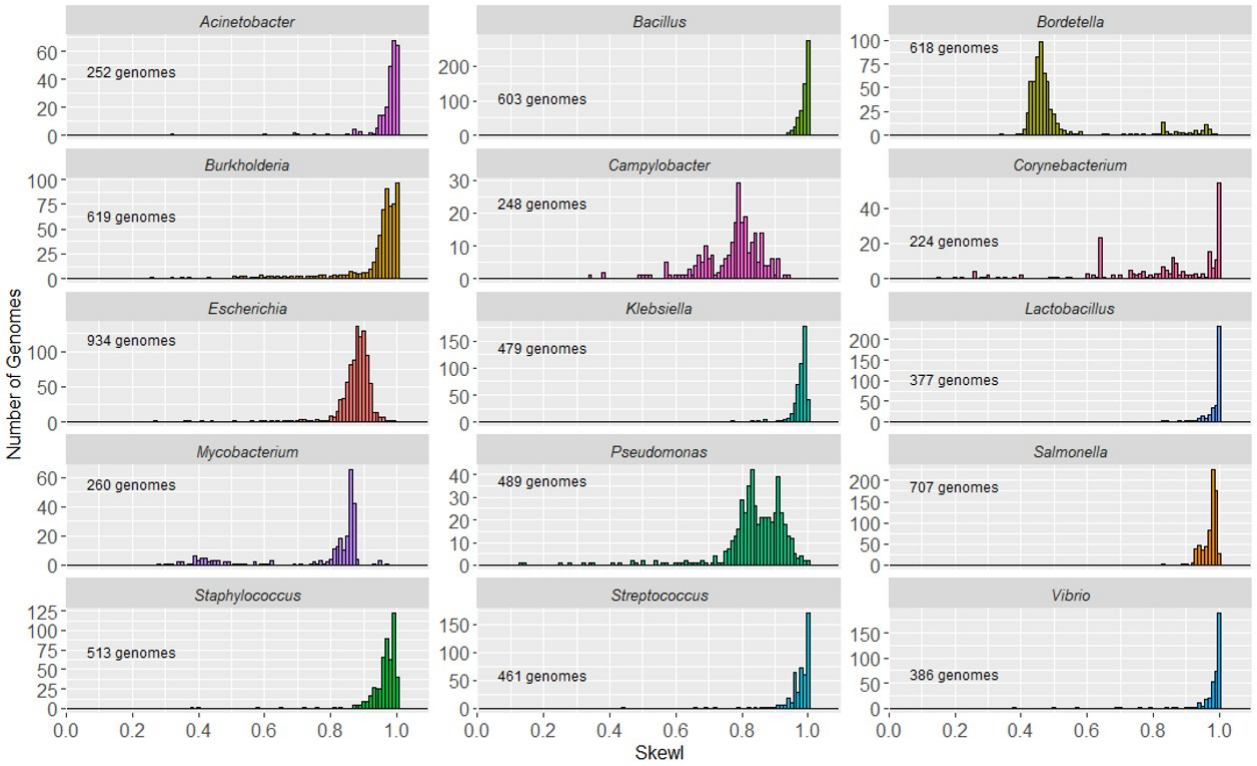
Skew index (SkewI) per genus. This figure shows the distribution of SkewI values for the 12 bacterial genera with the greatest number of fully sequenced genomes.

Overall, analysis of all bacteria revealed that most genomes have strong GC skew patterns, with relatively few having SkewI values less than 0.5 (S2 Fig). In order to isolate and analyze bacterial genomes with unusually low SkewI values, we separated the bacterial genomes by clades, revealing characteristic SkewI distributions for individual genera (Fig 2). For example, genomes from the genera of *Bacillus*, *Escherichia*, and *Salmonella* have consistently high SkewI values, with a mean close to 0.9. However, *Bordetella* genomes have far lower SkewI values, with a mean of 0.52. Additionally, while genomes in the *Klebsiella* and *Brucella* genera all have similar SkewI values (and therefore similar amounts of GC skew), genomes from the *Campylobacter* and *Corynebacterium* genera demonstrated much less consistent amounts of GC skew, with a wide range of SkewI values.

Given the differences between genera, we evaluated abnormalities in GC skew by setting a threshold for each genus that would allow us to flag genomes that might have assembly problems. For each genus with 10 or more genomes, we set a SkewI threshold at two standard deviations below the mean (S2 Table). If a genome’s SkewI exceeded the threshold, then we considered that bacterial genome to be within the expected range for that genera. However, if a genome’s SkewI was below the threshold, then we considered that genome to be possibly mis-assembled.

From our analysis, 161 genera of the total 1,148 analyzed contain 10 or more genomes. These 161 genera represent 12,846 of the 15,067 bacterial genomes analyzed, with 423 genomes having SkewI values below the threshold for their particular genus. Table 1 lists the SkewI statistics for the 12 bacterial genera with the greatest number of complete genomes.

**Table 1:** Average SkewI values for the 12 bacterial genera with the largest number of complete genomes. The threshold was set at 2 standard deviations below the mean.

Genus	Genome Count	Mean SkewI	SkewI St. Dev.	SkewI Threshold	Genomes Below Threshold	Mean GC-content (%)
Escherichia	934	0.8729	0.0620	0.7489	30	50.68
Salmonella	707	0.9682	0.0393	0.8896	15	52.15
Burkholderia	619	0.9323	0.1086	0.7151	39	67.42
Bordetella	618	0.5152	0.1474	0.2204	0	67.52
Bacillus	603	0.9848	0.04452	0.8957	10	41.31
Staphylococcus	513	0.9605	0.0538	0.8530	10	33.13
Pseudomonas	489	0.8359	0.1095	0.6170	20	63.09
Klebsiella	479	0.9746	0.03153	0.9115	17	57.23
Streptococcus	461	0.9743	0.0451	0.8840	12	33.44
Vibrio	386	0.9802	0.0559	0.8685	9	45.69
Lactobacillus	377	0.9799	0.0612	0.8574	11	42.99
Mycobacterium	260	0.7589	0.1730	0.4129	21	66.09
Acinetobacter	252	0.9715	0.0649	0.8418	7	39.37
Campylobacter	248	0.7714	0.0930	0.5853	13	30.98
Corynebacterium	224	0.8220	0.2001	0.4204	16	55.15

In order to investigate the genomes with SkewI values below the threshold, we focused on genome assemblies with accompanying read data that could be used to validate the assembly. Although there were 434 genomes with SkewI values below the threshold for their particular genus, 325 of these genome assemblies (75%) did not provide the reads used for assembly. 23 genome assemblies provided only short read data while 30 provided long read data. Only 56 of the 434 genomes (13%) listed both long and short reads used for genome assembly. For example, both the *Chlamydia* and *Corynebacterium* genera contained 16 genomes with low SkewI values relative to the expected SkewI for that genus. However, for both of these genera, all 16 genome assemblies did not provide any read data. We also were missing read data for the 11 *Lactobacillus* genomes below threshold and the 10 *Bacillus* genomes below the SkewI threshold. For the genomes and genera where read data was available, we identified several potentially mis-assembled *Escherichia* and *Burkholderia* genomes. Additionally, we were able to identify an interesting phenomenon in *Mycobacterium* genomes relating GC-Skew to GC-content. The following sections describes these findings.

### Escherichia

**Fig 3:**
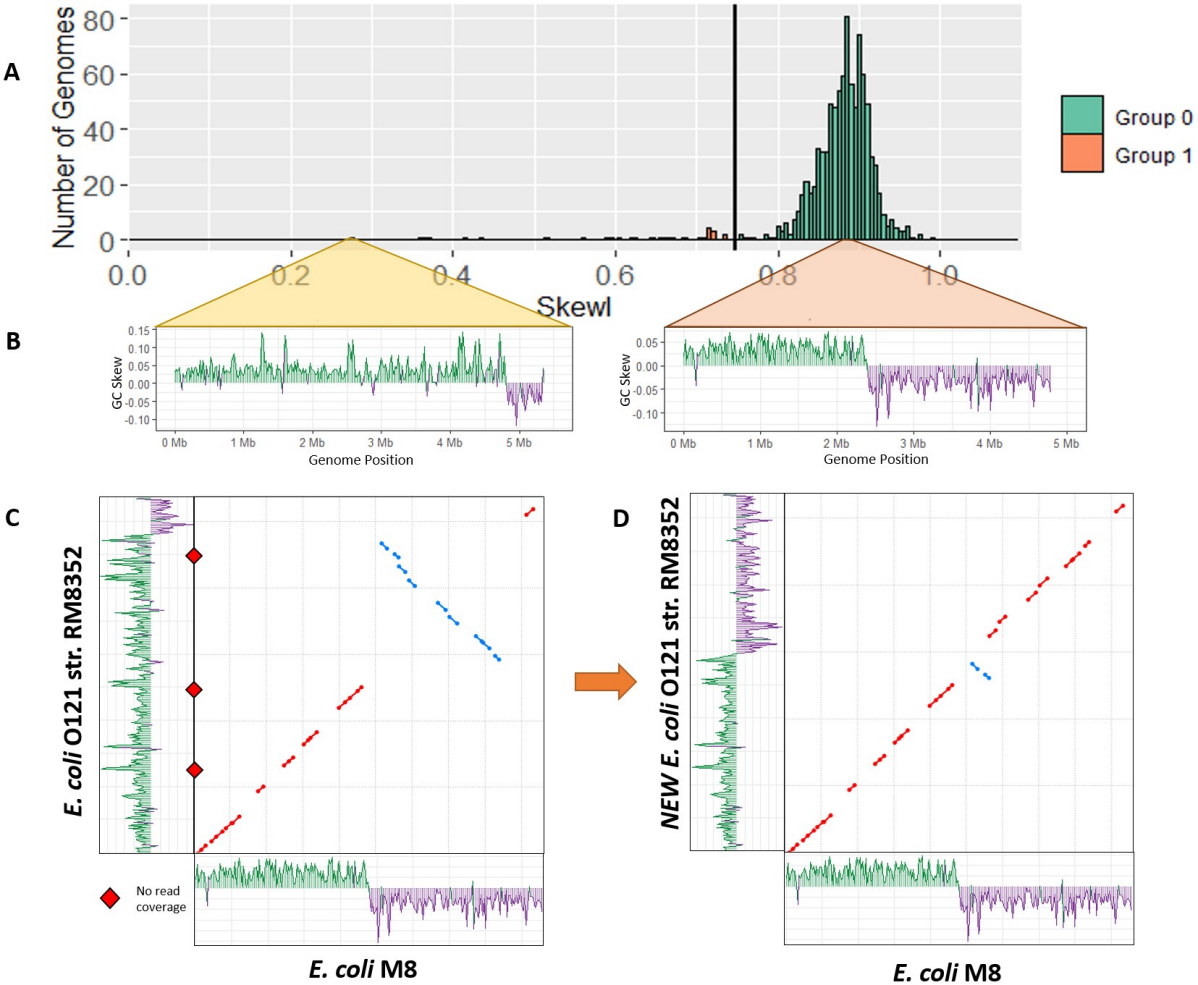
Escherichia skew index values. A) SkewI for all 934 *Escherichia* genomes. The threshold (vertical black line) is at 0.749. B) GC-skew plots for *Escherichia coli O121 strain RM8352* and *Escherichia coli M8*. *E. coli O121* has an unusually low SkewI of 0.275, while *E. coli M8* has a SkewI of 0.877, which is typical for this genus. C) Initial alignment between the two *E. coli* genomes revealed a large inversion. Alignment of the assembly reads revealed locations with no read coverage (red diamonds) *E. coli O121* at both ends of the inversion. D) Flipping the inversion in strain RM8352 produced a much more consistent alignment between the *E. coli* genomes (dot plot), and restored the GC skew plot to a more normal appearance (shown along the y axis).

For the *Escherichia* genus, RefSeq contains 934 complete genomes, with an average SkewI value of 0.87 and a threshold of 0.75 (Fig 3A). While the majority of *Escherichia* genomes had SkewI values above the threshold, one of them, *Escherichia coli O121 strain RM8352* (*E. coli O121*), had a SkewI of 0.275, which appeared far too low. In an effort to validate this assembly, we aligned the original raw reads back to the genome while also comparing *E. coli O121* to *Escherichia coli M8*, which has a more-typical SkewI of 0.877. Initial analysis of the GC-skew plots for both *E. coli* genomes revealed a clear difference between the genomes, as shown in Fig 3B. For *E. coli M8*, the GC skew plot shows that almost precisely half the genome has more Gs than Cs, and the other half has more Cs than Gs, as is typical for this species.

In *E. coli O121*, by contrast, a much larger portion of the forward strand has more Gs than Cs. We then aligned *E. coli O121* against *E. coli M8* (using used NUCmer [23]), revealing a large inversion in *E. coli O121* from position 2,583,081 to 4,963,263. Alignment of assembly reads to each genome using Bowtie2 [24] revealed gaps in coverage at the points flanking both ends of the inversion in *E. coli O121*, suggested that the assembly is incorrect in those regions (Fig 3C).

Because there were no reads supporting the inversion from 2,583,081 to 4,963,263 in *E. coli O121*, we replaced this sequence with its reverse complement and repeated our analysis. Our new *E. coli O121* genome has a SkewI of 0.838 with an evenly divided GC-skew plot (Fig 3D). Comparison of the new *E. coli O121* against *E. coli M8* shows a much more consistent 1-to-1 alignment between the two genomes, with only one small inversion remaining.

### Burkholderia

**Fig 4:**
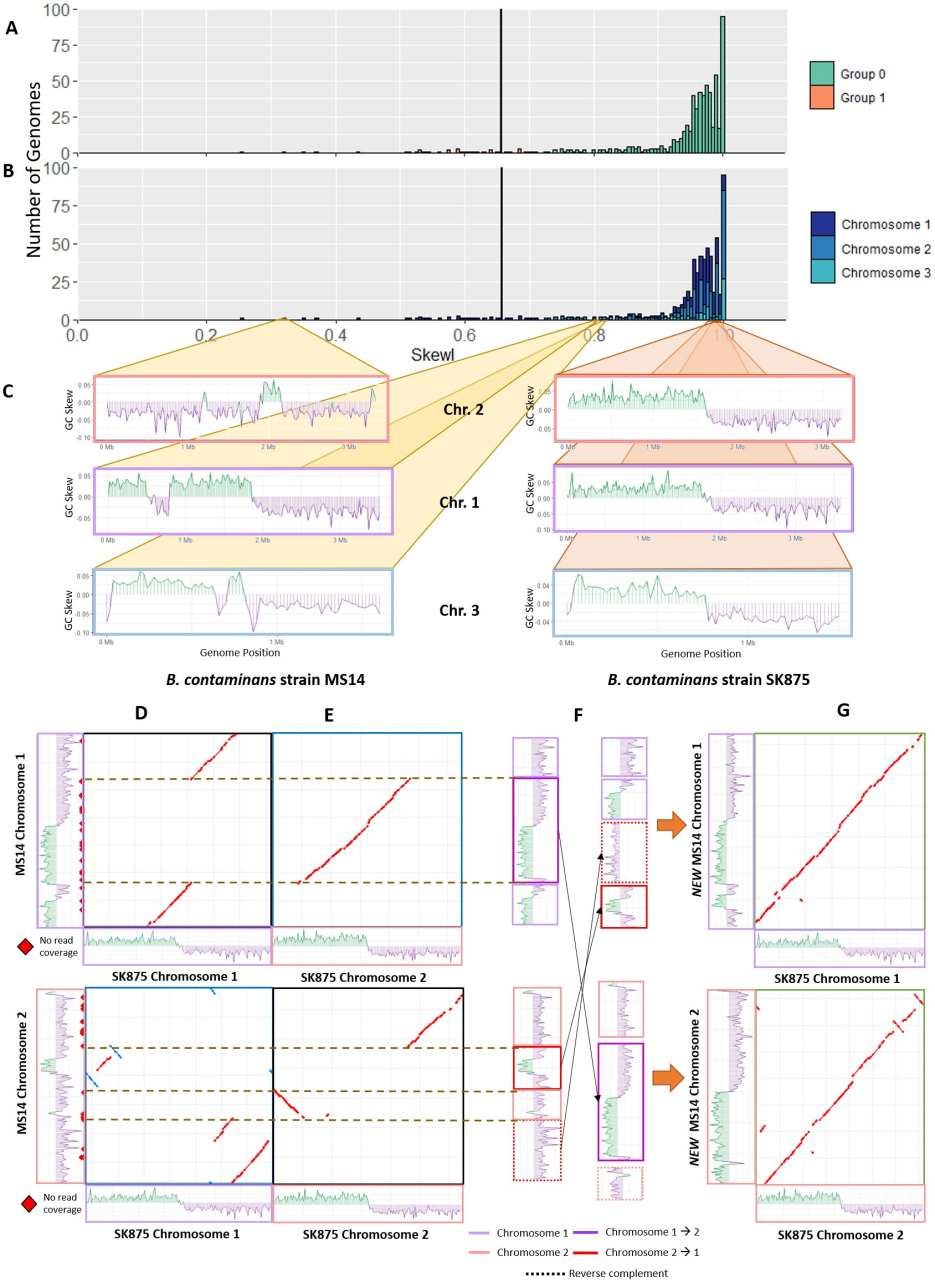
Burkholderia skew. A) SkewI for all 934 *Burkholderia* genomes. The threshold (vertical black line) is 0.715. B) SkewI colored by chromosome. C) GC-skew plots for all three chromosomes for *Burkholderia contaminans* strains MS14 (left) and SK875 (right). D) Alignments between MS14 and SK875 chromosomes 1 and 2. MS14 is shown on the y axis of each plot. E) Cross-chromosome alignments between MS14 and SK875 chromosome 1 and 2 reveal that a 1.7Mbp region of MS14 chromosome 1 actually belongs to chromosome 2. Similar matches in MS14 chromosome 2 suggest two regions that belong in chromosome 1. F) We rearranged and inverted the sequences of MS14 chromosomes 1 and 2 based on the alignments and GC-Skew plots. G) The final MS14 chromosomes alignment with those of *B. contaminans* SK875.

The *Burkholderia* genomes have a mean SkewI of 0.932 with a SkewI threshold of 0.715 (Fig 4A). Although there are 619 finished chromosomes from the *Burkholderia* genus, they represent only 270 individual organisms; each *Burkholderia* strain typically has 2-3 chromosomes. Fig 4B shows the SkewI distribution based on chromosome. There is no significant difference in SkewI between chromosomes.

Further analysis of the individual genomes with SkewI values below the threshold revealed significant differences between the SkewI values for the three chromosomes of *Burkholderia contaminans MS14*. Notably, chromosome 2 had a SkewI of 0.322 while chromosomes 1 and 3 had SkewIs of 0.869 and 0.909 respectively (Fig 4C). By comparison, the three chromosomes of a different strain, *Burkholderia contaminans SK875*, all had very high SkewIs of 0.978, 1.000, and 1.000.

Aligning the raw *B. contaminans MS14* assembly reads against the three chromosomes using Bowtie2 [24] revealed many locations with no read coverage, suggesting that the full read set used for the assembly was not available. We then aligned the *B. contaminans MS14* chromosomes against the same chromosomes for *B. contaminans SK875* and observed multiple large-scale disagreements between the chromosomes. While chromosome 3 from both strains aligned nearly perfectly, only 50% of chromosome 1 and 2 of MS14 aligned to the same corresponding chromosome of *B. contaminans SK875* (Fig 4D).

We then aligned chromosome 1 of *B. contaminans MS14* to chromosome 2 of *B. contaminans SK875* and vice versa and discovered that the sequences of *B. contaminans MS14* appeared mis-assembled (Fig 4E). Based on the differences in alignment and the GC Skew plots of *B. contaminans MS14*, it appears that the 1.7Mbp region of *B. contaminans MS14* chromosome 1 from 812,522 to 2,579,632 belongs to chromosome 2. Similarly, two regions from *B. contaminans MS14* chromosome 2 belong to chromosome 1. (We note here that it is possible that a very recent set of translocations and re-arrangements explains the anomalous SkewI value; however, the available data do not support that hypothesis).

Based on the chromosome alignments and GC-skew plots, we rearranged and inverted the individual *B. contaminans MS14* sequences as illustrated in Fig 4F. The final SkewI for these corrected chromosome 1 and chromosome 2 sequences were 0.872 and 0.966 respectively, both within the expected range. Additionally, realigning the new MS14 sequences against those of SK875 a far higher degree of synteny between the two genomes (Fig 4G).

### SkewI versus GC content and mycobacterium

**Fig 5:**
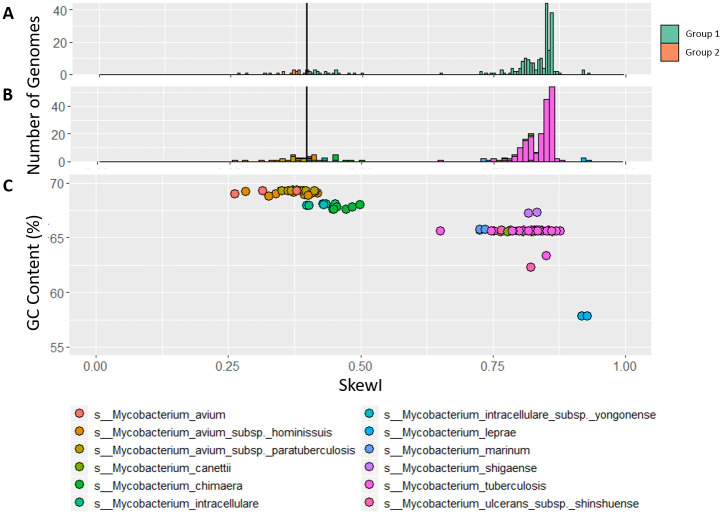
Mycobacterium skew index values. A) SkewI for 236 *Mycobacterium* genomes from 12 *Mycobacterium* species, all of which have multiple strains available in RefSeq. The threshold (vertical line) is at 0.413.) SkewI colored by species. C) Plot comparing GC Content (%) to SkewI, where each dot represents a different genome colored by species.

Analysis of the *Mycobacterium* SkewI distribution revealed a main peak at 0.85 and a smaller peak centered around 0.4 (Fig 5A). Due to the large standard deviation, the SkewI threshold was calculated to be 0.413, with 21 genomes falling below the threshold. However, upon investigation into the individual genomes, it appeared that all 21 of these genomes come from *Mycobacterium avium* and *M. avium* subspecies, suggesting that the SkewI values are not reflective of a mis-assembly but rather reflective of a different degree of skew in *M. avium* and possibly other species within the Mycobacteria.

We explored this hypothesis by re-plotting SkewI using different colors for each of the 12 species, as shown in Fig 5B. As the plot shows, the large peak centered around 0.85 mainly consists of the 179 *M. tuberculosis* genomes while the smaller peak mainly consists of the 27 *M. avium* genomes. Because *Mycobacterium* genomes have a high GC-content (%), we then plotted GC-content vs. SkewI for these same genomes (Fig 5C), revealing that for the *Mycobacterium* genus, higher GC-content results in a lower SkewI.

Although higher GC-content species within the *Mycobacterium* genus tend towards lower SkewI values, this evolutionary-based relationship [25] is not true across all bacterial clades. Upon analysis of the 12 bacterial genera with the greatest number of complete genomes, higher average GC-content does not necessarily reflect a low mean SkewI value (and vice versa, Table 1). For example, genomes in the *Mycobacterium*, *Burkholderia*, and *Bordetella* genera all have high GC-content (66%, 67% 68% respectively). However, while the average SkewI for *Mycobacterium* and *Bordetella* are relatively low (0.7589 and 0.5152), the average SkewI for *Burkholderia* genomes is at the higher end of the SkewI spectrum (0.9323). Similarly, the low GC-content genera of *Acinetobacter* and *Campylobacter*, (GC-content values of 39%, 31% respectively) have different mean SkewI values; *Campylobacter* genomes have an average SkewI of 0.77 while *Acinetobacter* genomes have an average SkewI of 0.97.

For a more in-depth analysis, we compared SkewI versus GC-content in S3 Fig. S3A Fig displays SkewI and GC-content for all 15,000+ RefSeq bacterial complete genomes while S3B Fig plots the mean SkewI and mean GC-content for every bacterial genus. However, analysis of both figures revealed no relationship between SkewI values and GC-content.

We then generated the same SkewI vs. GC-content figures for genomes in specific genera. S4 Fig shows the SkewI and GC-content distributions for genomes in the *Bacillus*, *Escherichia*, *Salmonella*, and *Burkhoderia* genera. While there is evidence that GC-content is conserved within species, there is no relationship between SkewI and GC-content for these genera. By comparison, S5 Fig shows similar SkewI/GC-content plots for *Mycobacterium* and *Bordetella*. For these two genera, there is some evidence that certain low GC-content species have higher SkewI values. However, while the patterns are more pronounced for *Mycobacterium*, there are some *Bordetella* species that follow this pattern (e.g. *Bordetella pertussis* and *Bordetella parapertussis*), there are also some *Bordetella* species that do not (e.g. *Bordetella flabilis*).

### Simulated mutations

Following our analysis of existing genomes and their SkewI values, we performed a set of simulation experiments to measure the sensitivity of the SkewIT method for detecting misassemblies of various sizes. First, we randomly selected 10 genomes belonging to each of the following species: *Bacillus thuringiensis* (SkewI threshold 0.896), *Salmonella enterica* (SkewI threshold 0.890), *Staphylococcus aureus* (SkewI threshold 0.853), *Escherichia coli* (SkewI threshold 0.759), and *Pseudomonas aeruginosa* (SkewI threshold 0.617). All selected genomes had SkewI values above the SkewI threshold for that genus.

For each genome, we simulated a misassembly error where a random subsequence, of length *k*% of the full genome length, is moved to another random location in the genome. We tested 12 different values of *k* = 2.5, 5, 7.5, 10, 12.5, 15, 17.5, 20, 22.5, 25, 27.5, 30 and for each value of *k*, we generated 100 randomly misassembled genomes and subsequently calculated the SkewI value of the misassembled genome. We then calculated the average number (across all 10 genomes for a given species) of misassembled genomes whose new SkewI values fell below the SkewI threshold for that genus.

**Fig 6:**
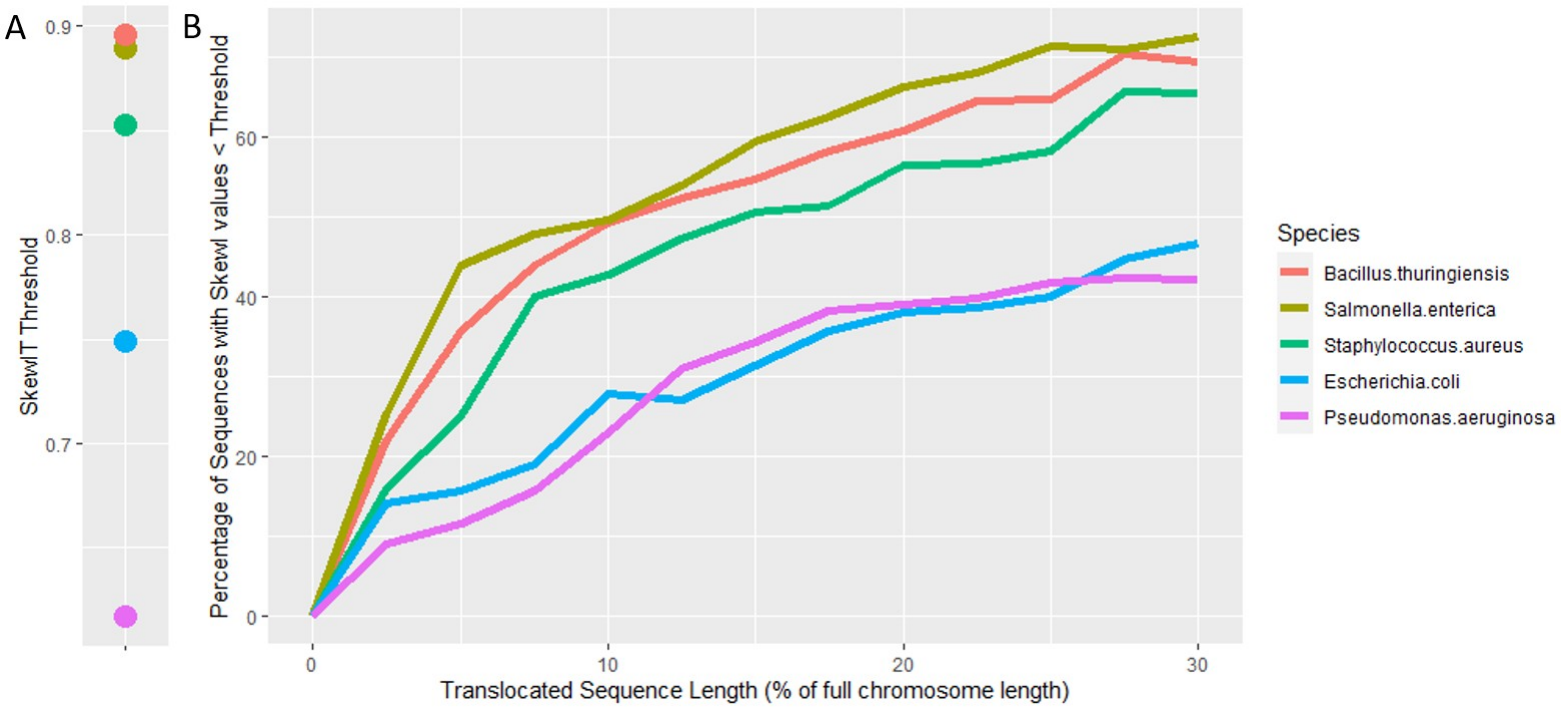
SkewIT sensitivity to misassemblies. In order to evaluate the sensitivity of the SkewIT method for detecting misassemblies, we first randomly selected 10 genomes from these species: *Bacillus thuringiensis*, *Salmonella enterica*, *Staphylococcus aureus*, *Escherichia coli*, and *Pseudomonas aeruginosa*. A) displays the SkewI threshold for each species. For each genome, we simulated 100 misassembled genomes by moving a random subsequence of length *k*% of the full genome length to another random location. This was repeated for 12 values of *k* ranging from 0 to 30, with 100 random misassemblies for each value of *k*. B) shows the average percentage of the misassembled genomes that had SkewI values below the threshold.

Fig 6 summarizes the results of this translocation experiment. Fig 6A shows the different SkewI thresholds for each of the tested species. Fig 6B displays the average proportion of misassemblies detected (i.e., those whose SkewI values fell below the threshold) for each value of *k*. As the length of the intentionally-misplaced sequence increases, the number of misassemblies detected increases. For example, moving a subsequence spanning only 5% of the full genome length yields a very small change in GC Skew. Approximately 20% of these misassemblies reduced the SkewI values sufficiently for the SkewIT method to detect the change. However, when long subsequences are misplaced, the GC Skew pattern is disrupted more, decreasing the SkewI value. For example, the SkewIT method detected 60% of misassemblies when 20% of the *Bacillus thuringiensis* genome was randomly moved to an incorrect location. However, if only 5% of the same genome was moved, then the SkewIT method detected the misassembly only 36% of the time. Comparisons between the various species also shows that the SkewI values of species with higher thresholds, such as *Bacillus thuringiensis* and *Salmonella enterica*, are more sensitive to genome modifications/misassemblies.

### SkewIT runtime and computational resources

Execution of the SkewIT code for all 15,000+ NCBI RefSeq bacterial genomes required 30 minutes, using 112Mb of RAM. For a single genome, the SkewIT code calculated SkewI within 1 second, using only 50Mb of RAM. All code is single-threaded and can process multi-FASTA files.

## Conclusion

Our SkewIT (Skew Index Test) provides a fast method for identifying potentially mis-assembled genomes based on the well-known GC skew phenomenon for bacterial genomes. In this study, we described and implemented an algorithm that computes a new GC-skew statistic, SkewI, and we computed this statistic across 15,067 genomes from RefSeq, discovering that GC skew varies considerably across genera. We also used anomalous values of SkewI to identify likely mis-assemblies in *Escherichia coli O121 strain RM8352* and in two chromosomes of *Burkholderia contaminans MS14*. We suggest that researchers can validate future bacterial genome assemblies by running SkewIT and comparing the resulting SkewI value to the thresholds in S1 Table. Genomes with SkewI values lower than the expected threshold should be further validated by comparison to closely-related genomes and by alignment of the original reads to the genome.

## Supporting information

S1 Text Text describing supplemental figures and the SkewIT Shiny App. (PDF)

S1 Fig SkewI comparisons for window sizes 10Kb, 15Kb, 20Kb, 25Kb, 30Kb. (TIF)

S2 Fig SkewI for all 15,067 complete bacterial RefSeq genomes. (TIF)

S3 Fig SkewI vs. GC content for bacterial RefSeq genomes. This figure compares SkewI to GC-content of each bacterial genome. A) displays each individual genome as a separate point, while B) displays the average SkewI vs. average GC-content for each bacterial genus. Points in both plots are colored by phylum.
(TIF)

S4 Fig SkewI vs. GC content for *Bacillus*, *Escherichia*, *Salmonella*, and *Burkholderia* genera. This figure compares SkewI to GC-content for four bacterial genera where no relationship between SkewI and GC-content is present. Axes in each plot are specific to the range of SkewI and GC-content values for genomes within that genus. Points are colored by species.
(TIF)

S5 Fig SkewI vs. GC content for *Mycobacterium* and *Bordetella*. This figure compares SkewI to GC-content for two bacterial genera where higher GC-content genomes tend towards lower SkewI values. Axes in each plot are specific to the range of SkewI and GC-content values for genomes within that genus. Points are colored by species.
(TIF)

S6 Fig SkewIT App: SkewI calculation and GC Skew Plot. The main panel in the application allows users to upload any FASTA file from which the program will generate a GC Skew plot and calculate the SkewI value for the FASTA sequence.
(TIF)

S7 Fig SkewIT App: Refseq Release 97 bacterial SkewI distribution. The SkewIT App allows users to explore the SkewI values across all bacteria in this tab, coloring the plot based on Phylum, Class, or other taxonomic groupings.
(TIF)

S8 Fig SkewIT App: Refseq Release 97 bacterial SkewI distribution. The SkewIT App allows users to explore the SkewI values across all bacteria in this tab, coloring the plot based on Phylum, Class, or other taxonomic groupings.
(TIF)

S1 Table Bacterial genomes SkewI. All 15,067 bacterial genomes are listed along with their calculated SkewI values. Additionally, this table lists the kingdom, phyla, class, order, family, genus, and species names/NCBI taxonomy IDs for each genome.
(XLSX)

S2 Table SkewI thresholds per genus. For all bacterial genera analyzed, we list the number of genomes, the average SkewI, and the SkewI standard deviation. For any genus with more than 10 genomes, we also include the threshold used to flag possible mis-assemblies, which is 2 standard deviations below the mean.
(XLSX)
