# Supplementary material for: SkewIT: The Skew Index Test for large-scale GC Skew analysis of bacterial genomes
Source: PLoS Comput Biol. 2020 Dec 4;16(12):e1008439. doi: 10.1371/journal.pcbi.1008439 (PMC7717575; doi:10.1371/journal.pcbi.1008439)
Supplement: S1 Text — (PDF) [file pcbi.1008439.s001.pdf]

Supplemental Information

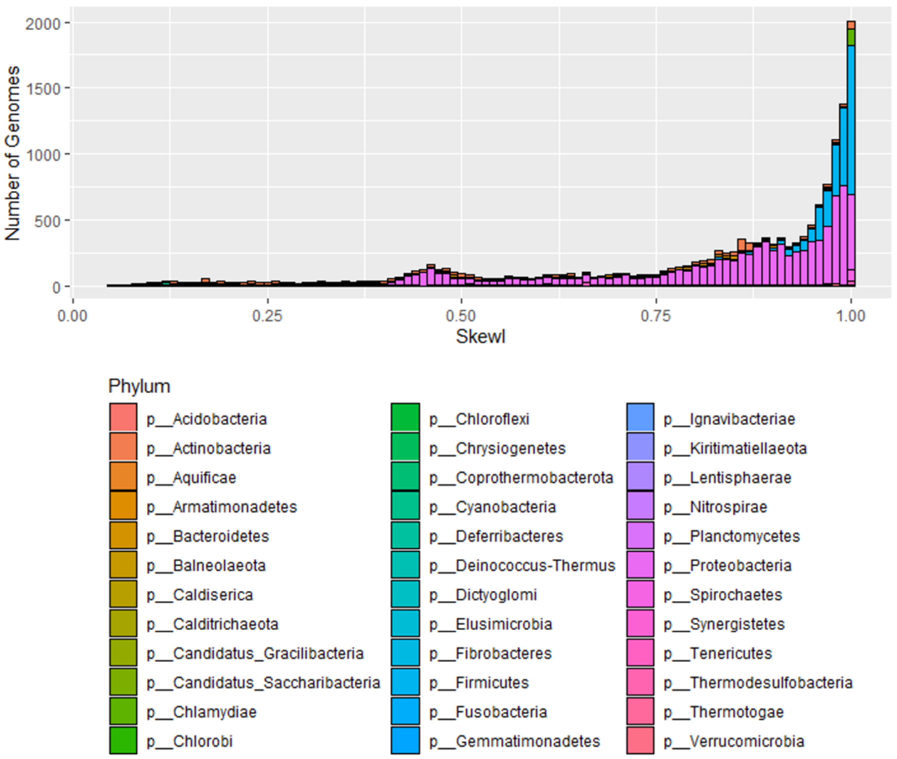

Suppl Fig 1. SkewI for all Refseq 97 Bacteria. This figure displays the full range of SkewI values for all complete bacterial chromosomes in Refseq Release 97, colored by phylum.

SkewIT Application

We have developed the SkewIT Application (available at `https://jenniferlu717.shinyapps.io/SkewIT/`) as an interactive web app which calculates SkewI and plots GC Skew from a user-provided bacterial genome FASTA file. **Supplemental Figure 2** displays the interface after a user has uploaded a FASTA file for sequence NZ_CP010191.1. The app displays the GC Skew plot and the SkewI value based on the user-selected window size and frequency. Frequency is the distance between the start of each window for which GC Skew is calculated. Users can regenerate the GC Skew plot and recalculate a new SkewI value by choosing new window size/frequency parameters. The default window size and frequency is 20kb. The app also allows users to scroll over the GC Skew plot to identify individual genome positions of interest.

The SkewIT app provides additional tabs for investigating the SkewI data generated in this paper for Refseq Release 97 Bacterial complete chromosomes. Under the

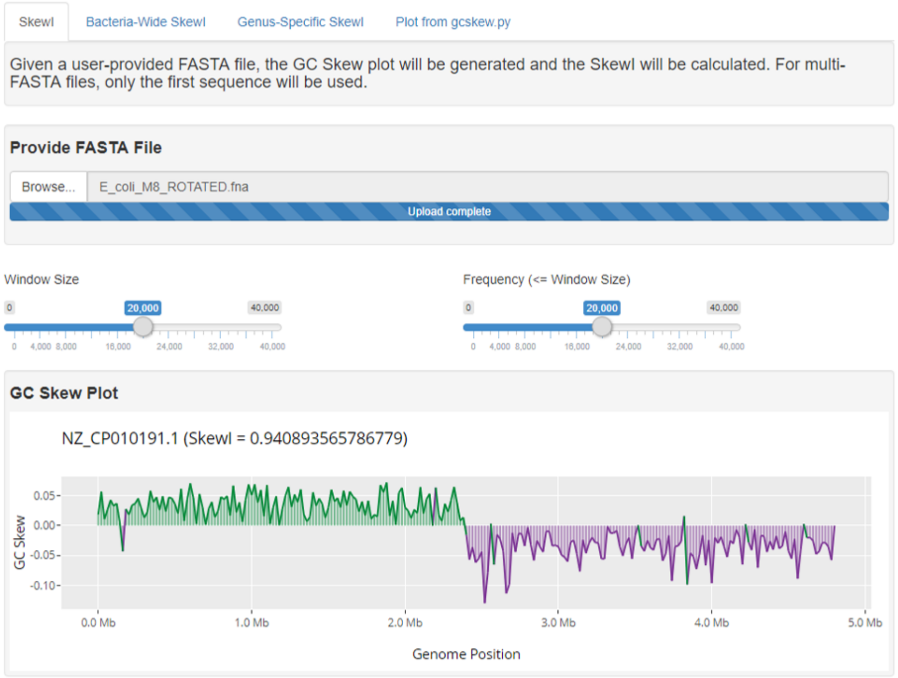

Suppl Fig 2. SkewIT App: SkewI Calculation and GC Skew Plot. The main panel in the application allows users to upload any FASTA file from which the program will generate a GC Skew plot and calculate the SkewI value for the FASTA sequence.

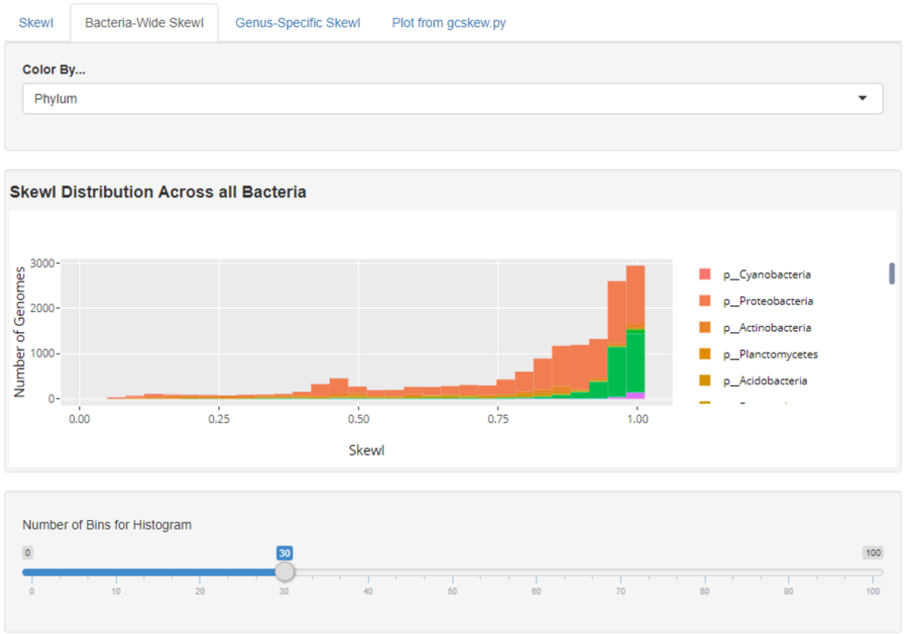

Suppl Fig 3. SkewIT App: Refseq Release 97 Bacterial SkewI Distribution The SkewIT App allows users to explore the SkewI values across all bacteria in this tab, coloring the plot based on Phylum, Class, or other taxonomic groupings.

"Bacteria-Wide SkewI" and "Genus-Specific SkewI" tabs, there are two different interactive interfaces for exploring SkewI values. First, for "Bacteria-Wide SkewI", as seen in **Supplemental Figure 3**, users can see the full range of Skew I values as colored by Phylum or other taxonomic levels of interest. The bins used in generating the SkewI histogram can be adjusted as well.

Finally, users can also investigate the SkewI values for individual genera in the "Genus-Specific SkewI" tab (**Supplemental Figure 4**). Following selection of a genus, the SkewI values will be displayed in two separate plots: as a histogram and as a dot plot. The dot plot allows additional investigation into SkewI values by plotting each chromosome as a single point, grouped by species within that genus. Scrolling over individual points will show the SkewI value for that genome and the sequence ID associated with that SkewI value.

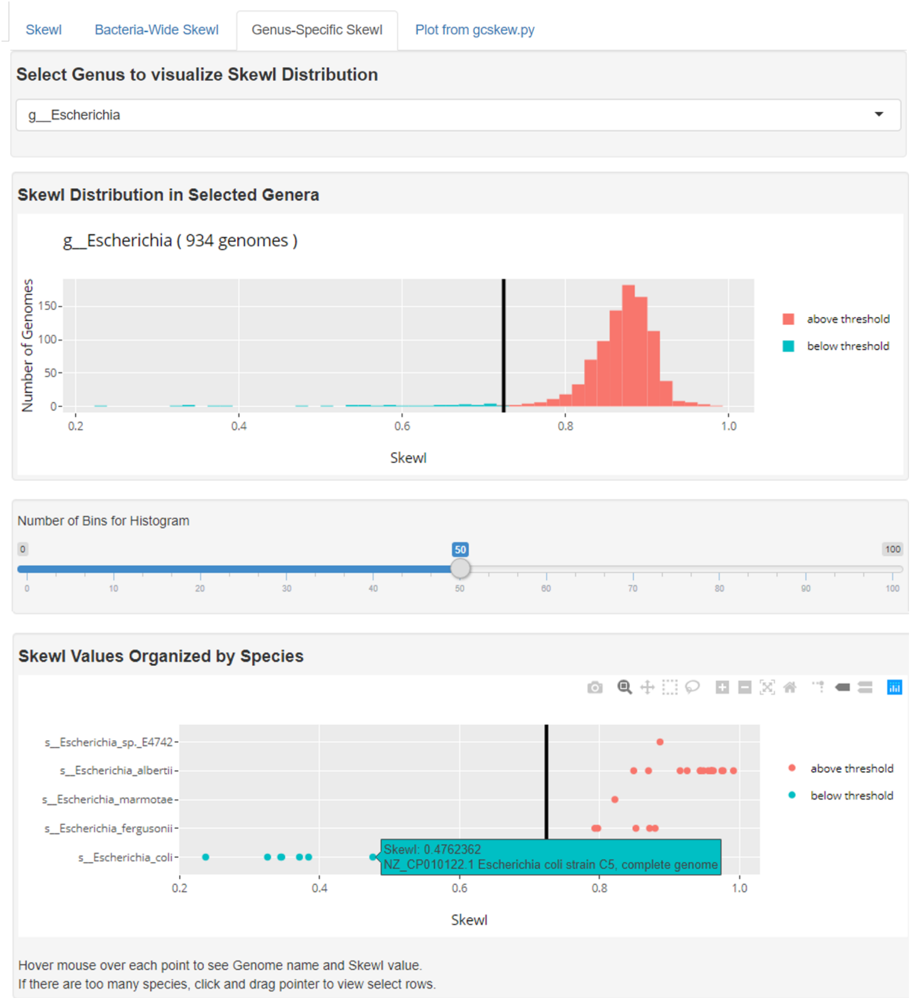

Suppl Fig 4. SkewIT App: Refseq Release 97 Bacterial SkewI Distribution The SkewIT App allows users to explore the SkewI values across all bacteria in this tab, coloring the plot based on Phylum, Class, or other taxonomic groupings.
