# Supplementary figures and images for: SkewIT: The Skew Index Test for large-scale GC Skew analysis of bacterial genomes
Source: PLoS Comput Biol. 2020 Dec 4;16(12):e1008439. doi: 10.1371/journal.pcbi.1008439 (PMC7717575; doi:10.1371/journal.pcbi.1008439)

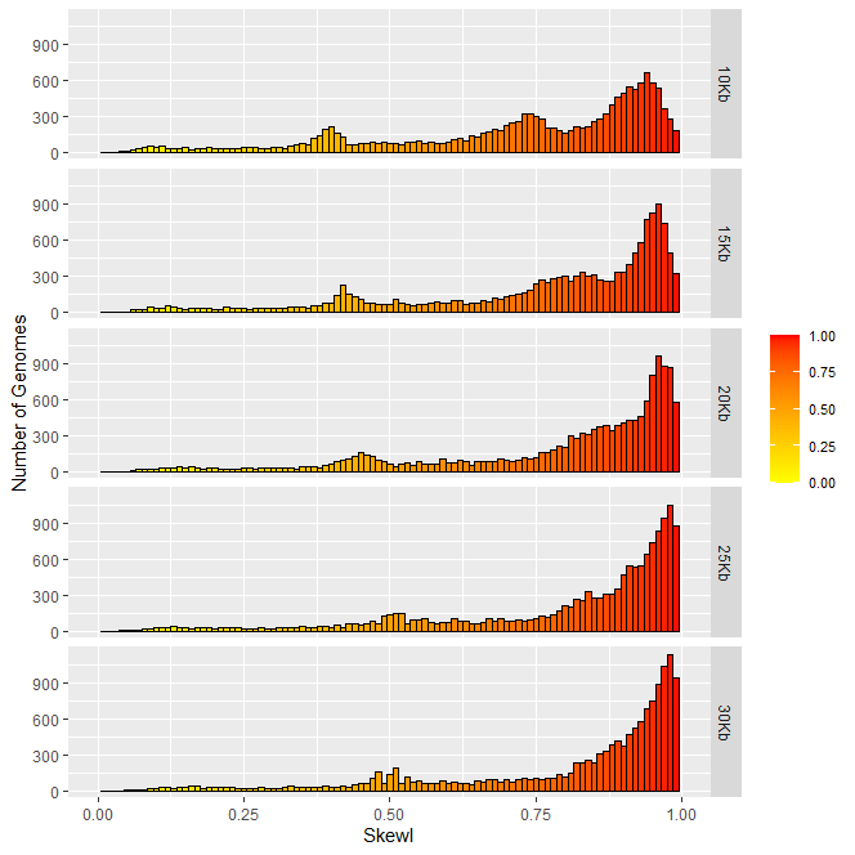

Supplement: S1 Fig — (TIF) [file pcbi.1008439.s002.tif]

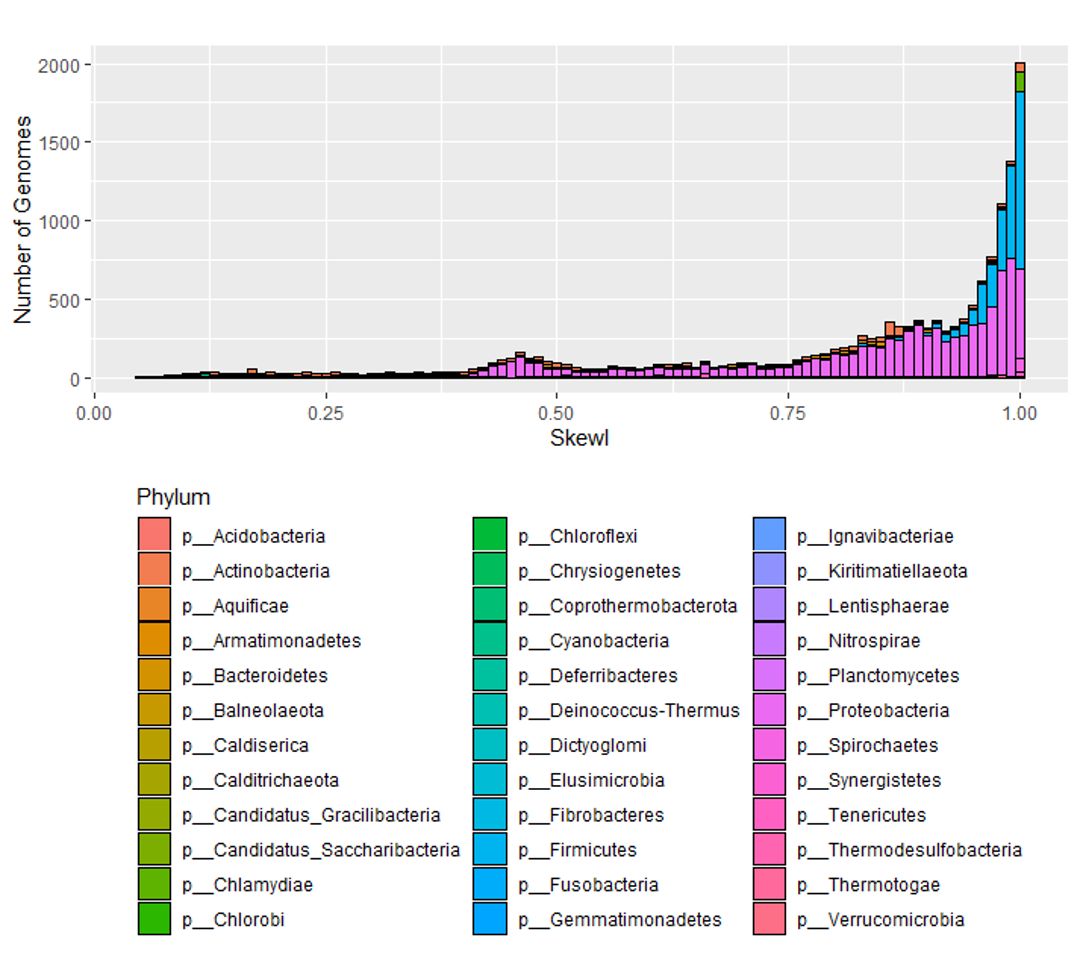

Supplement: S2 Fig — (TIF) [file pcbi.1008439.s003.tif]

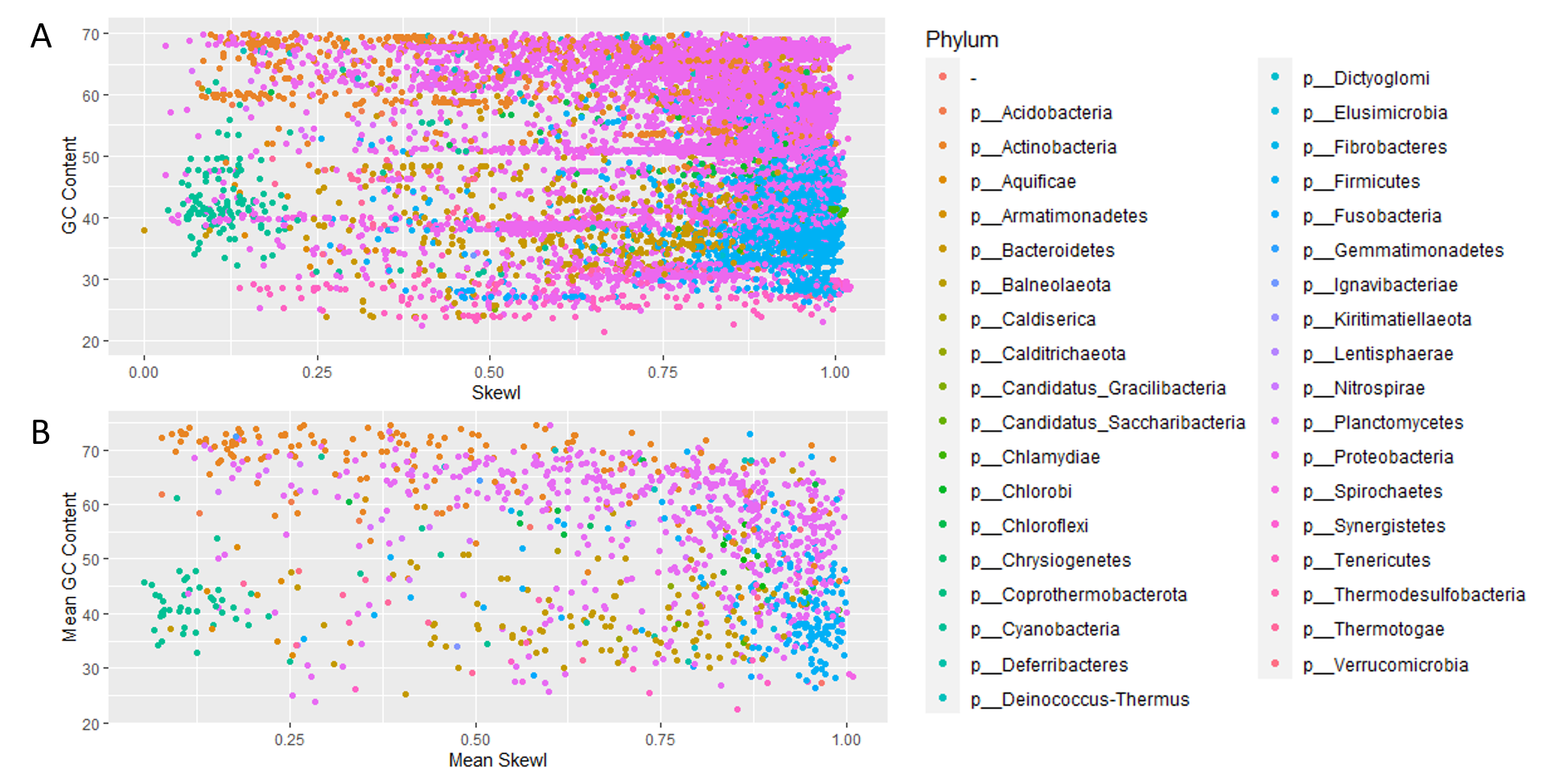

Supplement: S3 Fig — This figure compares SkewI to GC-content of each bacterial genome. A) displays each individual genome as a separate point, while B) displays the average SkewI vs. average GC-content for each bacterial genus. Points in both plots are colored by phylum. (TIF) [file pcbi.1008439.s004.tif]

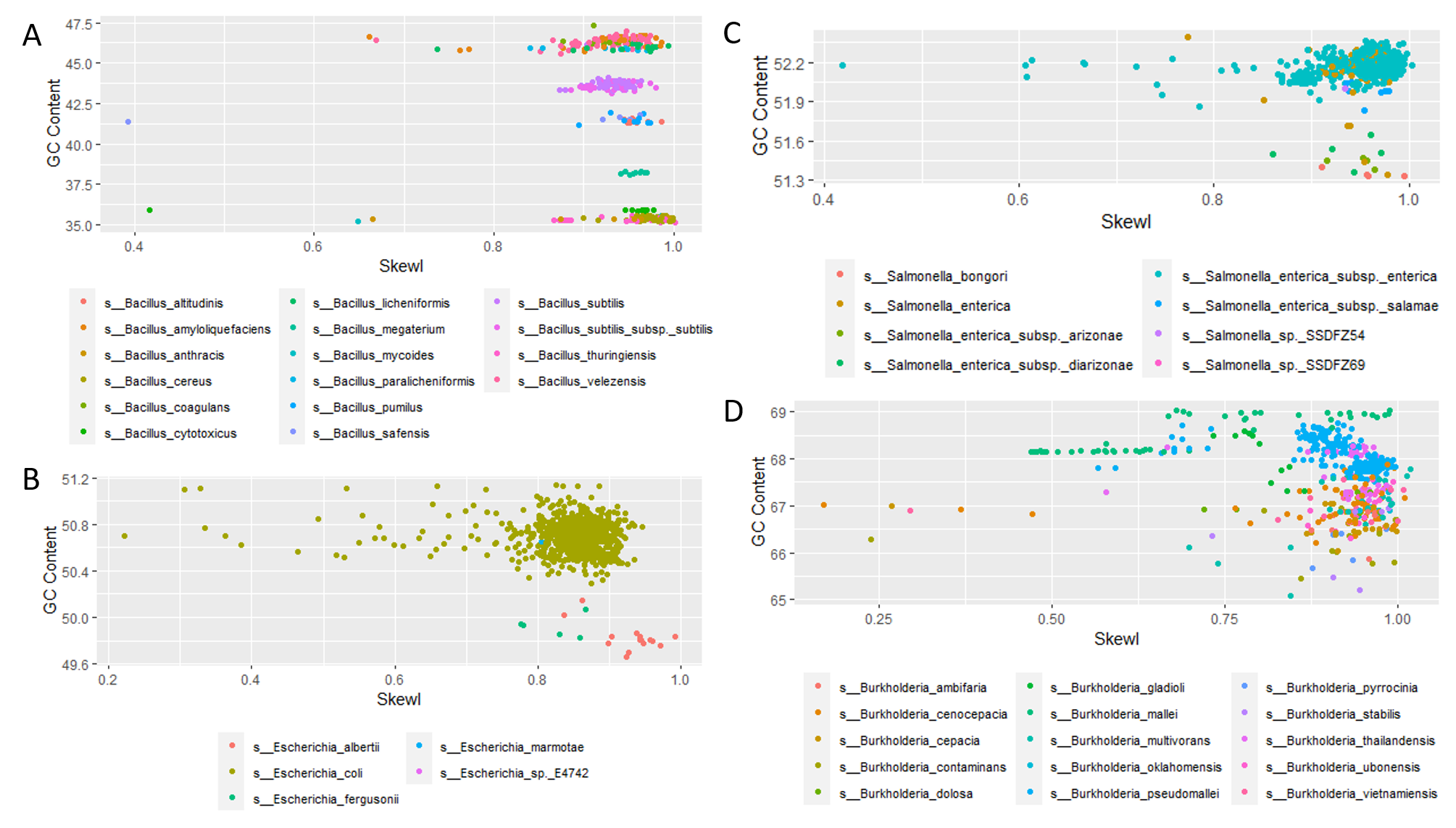

Supplement: S4 Fig — This figure compares SkewI to GC-content for four bacterial genera where no relationship between SkewI and GC-content is present. Axes in each plot are specific to the range of SkewI and GC-content values for genomes within that genus. Points are colored by species. (TIF) [file pcbi.1008439.s005.tif]

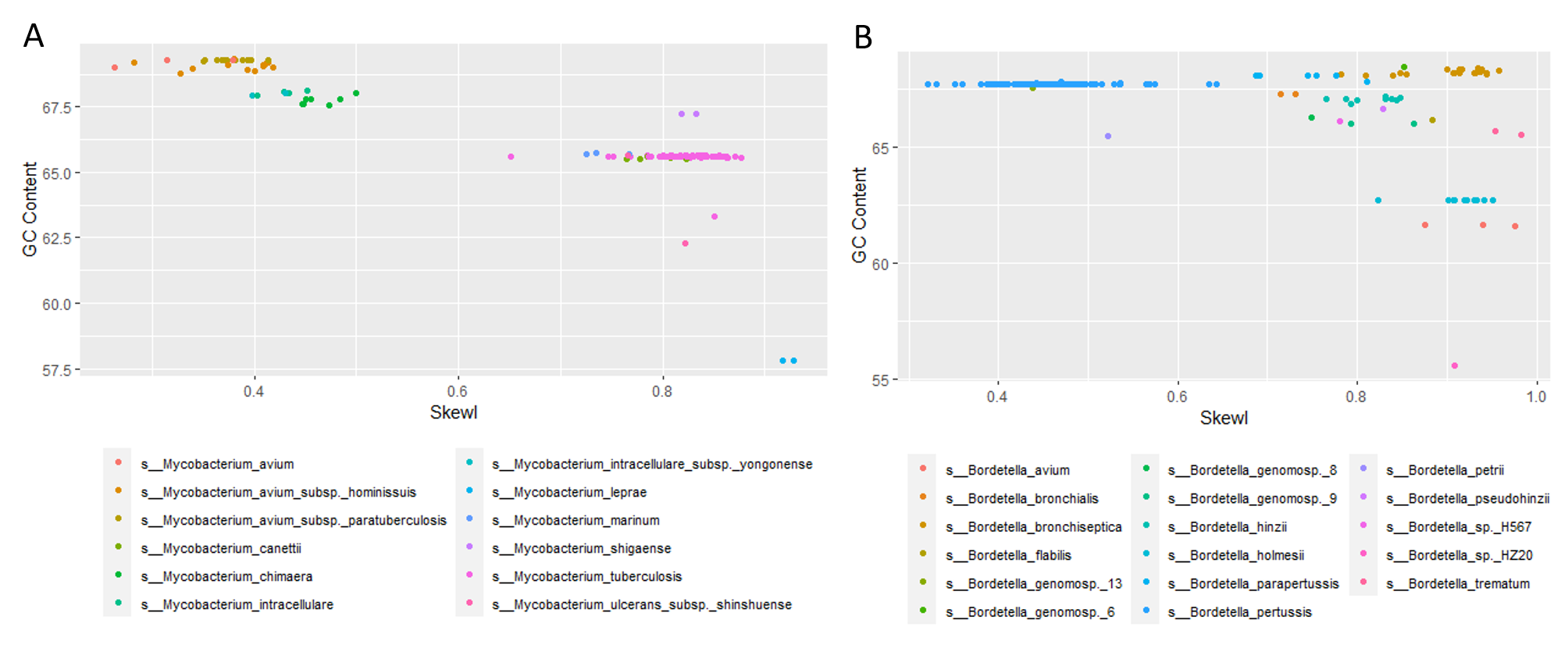

Supplement: S5 Fig — This figure compares SkewI to GC-content for two bacterial genera where higher GC-content genomes tend towards lower SkewI values. Axes in each plot are specific to the range of SkewI and GC-content values for genomes within that genus. Points are colored by species. (TIF) [file pcbi.1008439.s006.tif]

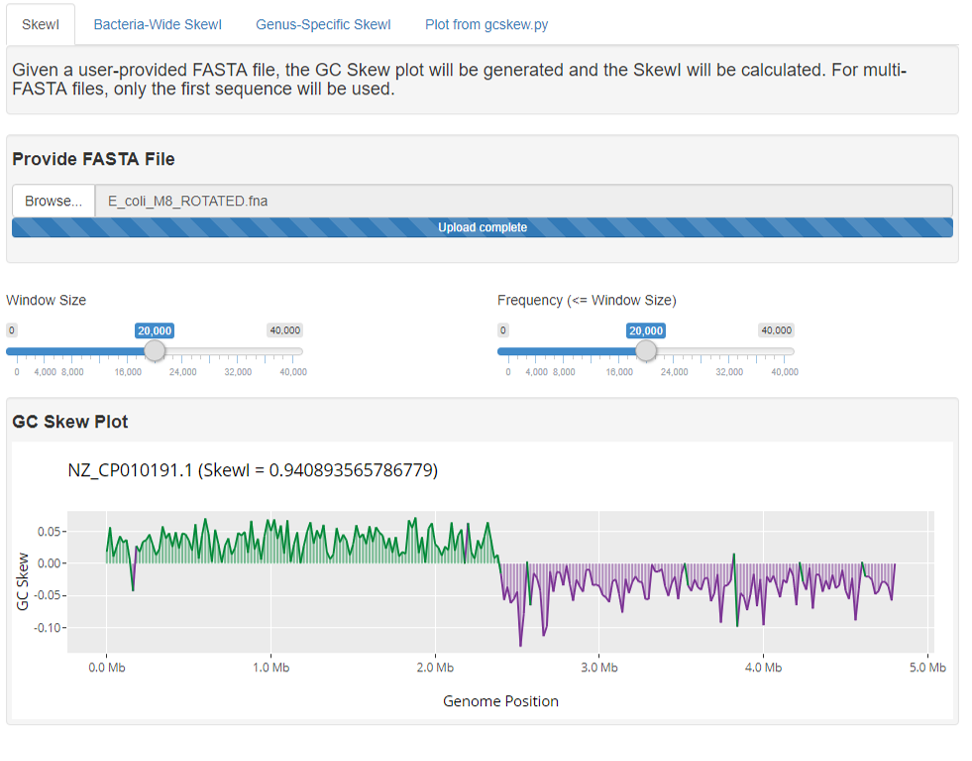

Supplement: S6 Fig — The main panel in the application allows users to upload any FASTA file from which the program will generate a GC Skew plot and calculate the SkewI value for the FASTA sequence. (TIF) [file pcbi.1008439.s007.tif]

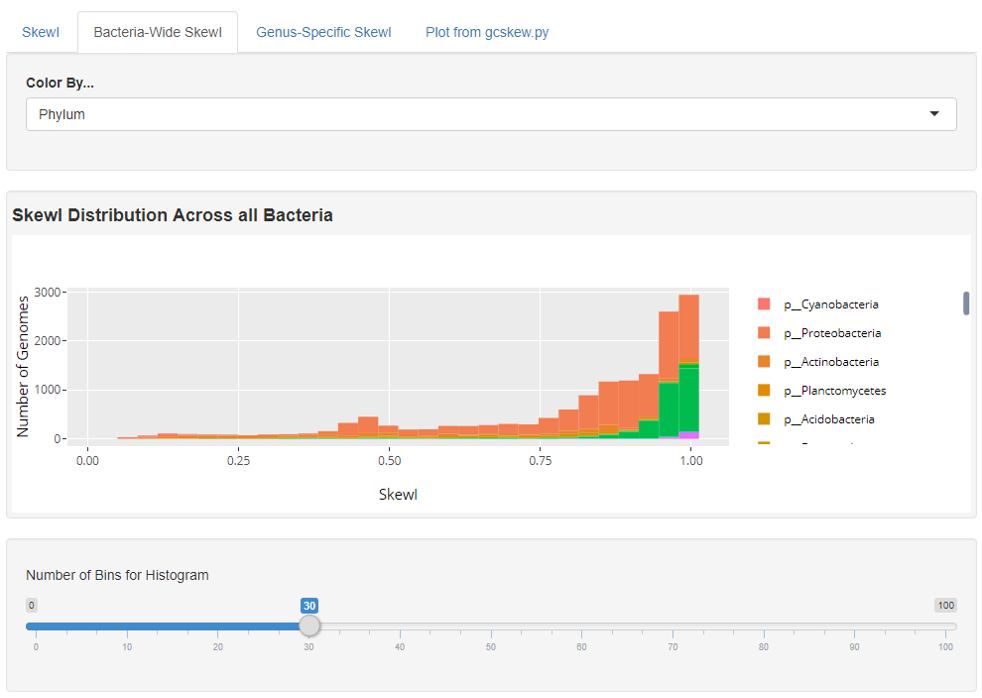

Supplement: S7 Fig — The SkewIT App allows users to explore the SkewI values across all bacteria in this tab, coloring the plot based on Phylum, Class, or other taxonomic groupings. (TIF) [file pcbi.1008439.s008.tif]

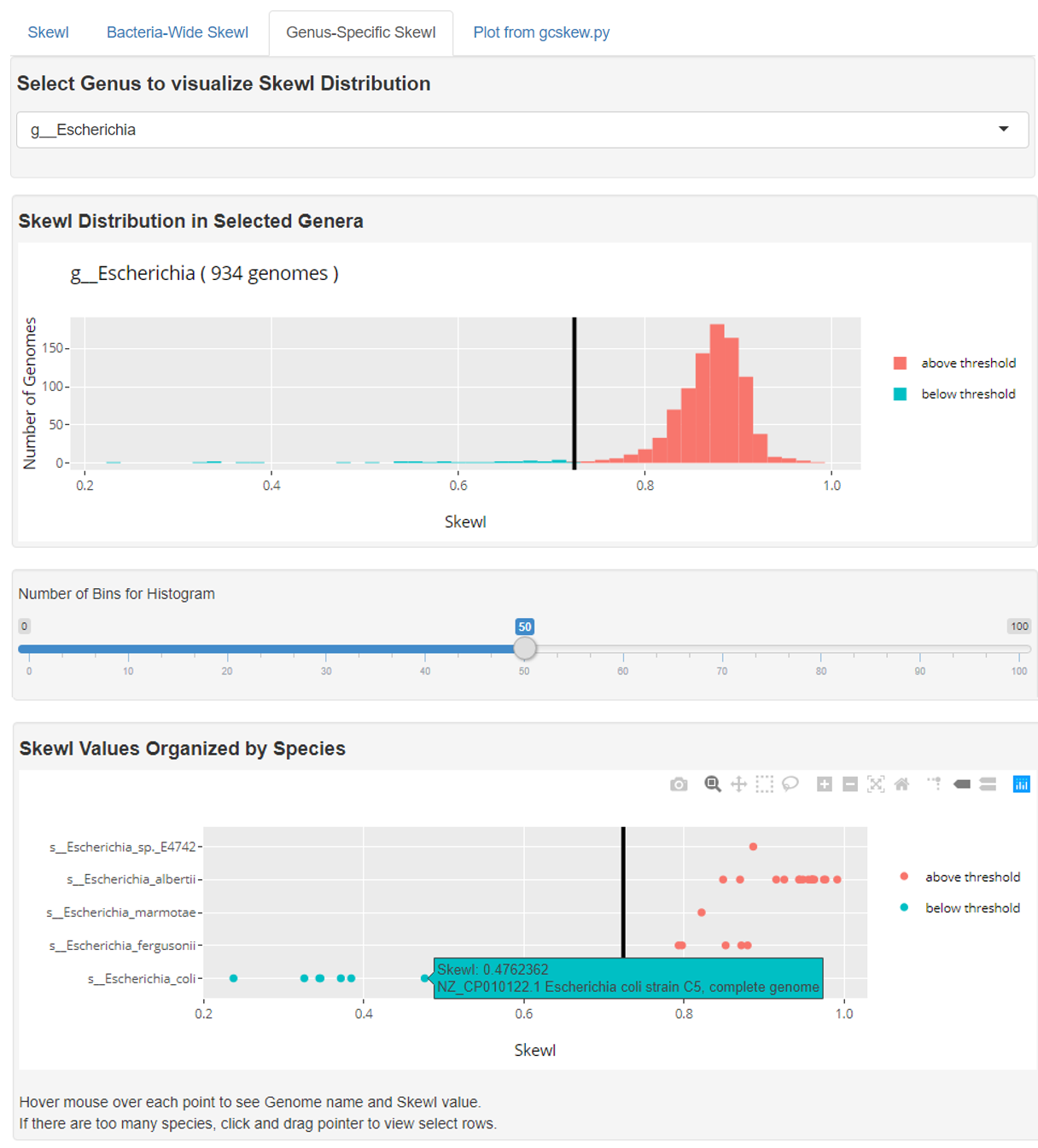

Supplement: S8 Fig — The SkewIT App allows users to explore the SkewI values across all bacteria in this tab, coloring the plot based on Phylum, Class, or other taxonomic groupings. (TIF) [file pcbi.1008439.s009.tif]
